# Social developmental delays among 3 to 6 year old children in preschools in German social hotspots: results of a dynamic prospective cohort study

**DOI:** 10.1186/s12887-020-02128-3

**Published:** 2020-05-13

**Authors:** Josefin Biermann, Marco Franze, Wolfgang Hoffmann

**Affiliations:** Institute for Community Medicine, Department Epidemiology of Health Care and Community Health, University Medicine Greifswald, Ellernholzstraße 1-2, 17487 Greifswald, Germany

**Keywords:** Early prevention, Preschools, Children aged 3 to 6 years, Social skills, Developmental delays, School-readiness, Prevalence, Risk factors

## Abstract

**Background:**

Social skills are valid predictors for school readiness and subsequent school success. The federal state law for child day-care and preschools in Mecklenburg-Western Pomerania, a federal state in Germany, provides additional funds for the targeted and individualized promotion of social developmental delays for children in preschools in social hotspots. The law grants additional funds to eligible preschools, provided that each child’s development is documented with a standardized, objective and valid screening instrument.

**Methods:**

To monitor the development and to detect social developmental delays, the preschools involved use the “Dortmund Developmental Screening for Preschools” (DESK 3–6). For the prevalence and risk factors, data of 5595 children aged 3 to 6 years from these preschools were analyzed.

**Results:**

9.6% of the children show reasonable findings in their social development; for a further 6.1% the results were inconclusive. Sex, presence of chronic diseases or disabilities and reasonable findings in the domains motor development and language and cognition were risk factors in terms of social development across all age groups.

**Conclusions:**

The federal state law is a good example for the implementation of a standardized monitoring of the development of children. With the help of this screening instrument, prevention activities to reduce the prevalence of developmental delays can be conducted in early childhood. Early preventive activities should take into account the reported risk factors for the social development.

**Trial registration:**

German Clinical Trials Register, ID: DRKS00015134, Registered on 29 October 2018, retrospectively registered.

## Background

The long-term study in Germany, the KiGGS study by the Robert Koch Institute (German Health Interview and Examination Survey for Children and Adolescents KiGGS Wave 2), provides representative nationwide data on young people’s health in Germany. By using a screening questionnaire (Strengths and Difficulties Questionnaire, SDQ) including sub-scales for emotional symptoms, peer relationship problems, conduct problems and hyperactivity/inattention, the nation-wide results show that 20.9% of the 3–5-year-olds (95%-confidence interval: 17.5–24.7) are affected by psychopathological problems and psychosocial impairments [1]. The results of the KiGGS Wave 2 also show that children from families with a lower socio-economic status are more affected by mental health problems than children from families with a high or middle socioeconomic status [1]. Other research also found out that children who grow up in poverty more frequently have delays in social and communication skills. In addition, these children more often display behavioral problems [2], lower social skills – especially self-regulation skills [3, 4] and reduced school-readiness [5].

Moreover, gender differences were found in the KIGGS Wave 2, e.g. boys show a higher risk for behavioral disorders, hyperactivity and peer-problems, whereas girls have a higher risk for emotional problems [6]. Several studies have shown evidence for a link between gender and children’s development [3, 6, 7].

Our investigation was based on a pilot-study, which revealed for 15.4% of the children tested at preschools that there were reasonable findings with regard to their social development, while the results for another 7.7% were inconclusive [3]. Further risk factors include linguistic and cognitive developmental delays and male gender [3]. Language skills are associated with social-emotional competencies, the ability to comply with demands and to build up positive relationships [2, 8, 9]. Language skills are necessary for social interactions. In consequence, children with specific language impairments are more often at risk for developmental delays in their social development [10].

In addition, in every situation or activity linguistic, cognitive, motor and social-emotional skills were promoted in a parallel way. For example, while playing soccer the children have to follow rules, have to run and have to communicate with their team [11]. In preschools, social behavior can be tested in and through sport activities with others. In addition, pedagogically guided and structured exercises offer enable specific situations in which taking and negotiating roles, agreeing on rules, making contact and cooperative behavior are relevant [11].

Previous research show that effective interventions provide the chance to reduce the school readiness gap which is associated with socioeconomic disadvantage [4]. For example, in Preschool PATHS, a randomized clinical trial evaluating an adaptation of the Promoting Alternative Thinking Strategies curriculum (PATHS) for preschool-age children in Head Start, 287 children were followed for 1 year, with skills assessed at the beginning and end of that year. The evaluation showed that children in the intervention group had higher emotion knowledge skills and were rated by parents and teachers as more socially competent compared to control peers [12].

### Social skills as valid predictors for school-readiness and -success 

There is a strong evidence that social skills can be considered as valid predictors for school- readiness and subsequent success at school [2, 13]. In general, children with behavioral problems and less social skills have problems to control their emotions and have less efficient social problem-solving strategies [8, 14]. Social skills are associated with a cooperative and successful interaction with peers and more supporting friendships [2, 7]. Furthermore, children with lower social skills have on average less empathy and self-regulation skills [5].

Socially competent children are able to pay more attention to their academic tasks, are able to plan better and to benefit more from instructions by teachers [2, 7]. Children with lower social skills tend to participate less in class, are rather non-compliant with rules and less accepted by peers and teachers [2, 5, 15]. One’s school career has been shown to be dependent on learning motivation, learning behavior and learning problems [16]. All of these factors are associated with social skills and, hence, disorders in this domain are negatively associated with one’s school career and even more so with success at school [2, 14, 16, 17]. On the other hand, social-emotional skills are important predictors of school achievement [2].

Children at risk of deficits in their social development have a higher risk of ruining relationships in the future and also of academic failure and violence [18]. A recent study of the OECD confirms that social-emotional skills are the most predictive skills of success in a wide range of important life outcomes, e.g. academic achievement, job performance, occupational attainment, health, longevity or personal and societal well-being [19]. Furthermore, a lack of these skills regularly correlated with unfavorable long-term outcomes such as an increased chance of unemployment, divorce, poor health, criminal behavior and imprisonment [19–21].

### Day-care in Germany

In Germany, preschools (in Germany called *“Kindergarten”*) are institutions for early childhood education and care for young children from age three to school entry. According to § 22 SGB (Social Code) VIII, the institution preschool has the aim to

- promote the development of every child into a responsible and sociable personality,
- support and supplement the family care and education and
- help the parents to arrange their work and parenting.

In preschools in Mecklenburg-Western Pomerania there are about 13.7 children per preschool-teacher, that is 4.5 children more than in Germany as a whole (1:9.2, [22]).

Due to the high utilization rate of preschools in Mecklenburg-Western Pomerania (in 2017: 0-to-3-year-olds: 56%, 3-to-6-year-olds: 95.2% [23]) preventive activities implemented in this setting have the chance to reach most of the children in Mecklenburg-Western Pomerania, including children with a lower socioeconomic status, without stigmatization.

The time spent in preschools is the period in which significant social skills are developed [17]. Prior to school entry, cognitive abilities and cognitive control (attentional performance and task persistence) have a great predictive power of future achievement outcomes, while prosocial behavior positively influences the learning motivation and the self-concept. Therefore, this setting provides a chance to integrate prevention activities to promote social skills. Moreover, a preschool is attended by most children (for at least 3 years) and therefore provides a relevant setting for long-term prevention activities [24].

Preventive activities can reduce social disparities and improve equal opportunities for school-readiness [5]. An appropriate promotion of competences requires a systematic and standardized observation and documentation of developmental dynamics [25]. Therefore instruments for developmental monitoring have become more important [13]. Sinzig & Schmidt [26] demand that preschool teacher should monitor the children’s development.

### The federal state law for child day-care and preschools in Mecklenburg-Western Pomerania

The federal state law for children’s day-care and preschools in Mecklenburg-Western Pomerania is designed to reduce social inequalities [27] by focusing on preschools in social hotspots. According to a definition of social hotspots from 1979, social hotspots in Germany are areas in which factors that determine the living conditions of their residents negatively occur more frequently. Especially, these factors influence the development opportunities of children and adolescents negatively [28].

The federal state law provides additional financial funds for the individual as well as group support for children with developmental delays in their motor, linguistic-cognitive or social development.

The federal state law for children’s day-care and preschools in Mecklenburg-Western Pomerania selects the preschools as follows: the youth welfare offices of each region in Mecklenburg-Western Pomerania determine the specific amount of preschool-fees that was covered by the state for each preschool. Subsequently, those in charge of preschools with an amount above the average are informed about the opportunity to receive additional funding according to the law (at least an annual amount of EUR 20,000 for preschools attended by < 50 three to 6 year olds, or at least an annual amount of EUR 40,000 for preschools attended by ≥50 three to six year olds, respectively) – the participation is voluntary for the preschools [29].

Mandatory criteria for claiming these benefits and funds from the State of Mecklenburg-Western-Pomerania is an annual application of a valid, standardized, objective, and reliable developmental screening instrument to monitor the development and to detect developmental delays (“Dortmund Developmental Screening for Preschools DESK 3-6”) [30, 31]. Another mandatory criterion for claiming additional funds is the participation in a scientific evaluation within the framework of the legislation for a standardized, objective, and valid assessment of developmental delays. Based on the result of the screening instrument, the targeted and individualized interventions of children at risk take place in the preschool [32].

### Purpose of the study

For the initiation of early individual intervention strategies, it is important to monitor the development and to detect developmental delays early on. For the planning and implementation of early prevention both valid prevalence rates and possible risk factors are essential [26]. The present paper reports the age-specific prevalence rates of developmental delays with regard to the social development of socially disadvantaged children aged from 3 to 6 years old in a total of 90 preschools in Mecklenburg-Western Pomerania. Further, we analyze the relationship between potential risk factors and the social development using a large sample size. The results help define and plan evidence-based intervention strategies in Mecklenburg-Western Pomerania.

## Method

### Study design

The evaluation of the federal state law for child day-care and preschools in Mecklenburg-Western Pomerania started in 2011 and is designed as a dynamic prospective cohort study.^1^ In 2020, 162 preschools participate in the evaluation. Based on the total number of preschools in Mecklenburg-Western Pomerania, *n* = 1082, this reflects a proportion of 15.2%.

### Study region

Mecklenburg-Western Pomerania is a rural state in Germany with an area of 23,293.73 km^2^ and a total population of 1,610,674 [33, 34]. In 2016, the population density was 69 people/ km^2^ [34]. Mecklenburg-Western Pomerania has a high poverty rate (unemployment rate in May 2018: 7.7% (Mecklenburg-Western Pomerania) vs. 5.1% (whole of Germany) [35].

In 2018 56.4% of the 0- to 3 year-olds in Mecklenburg-Western Pomerania (Germany: 33.6%), and 94.9% of the 3- to 6-year-olds in Mecklenburg-Western Pomerania attended a preschool (Germany: 93%) [23]. The preschools involved are located in social hotspots in cities (e.g. Wismar, Schwerin, Greifswald, Stralsund) and rural areas in the state.

### Instrument

The federal state law for child day-care and preschools in Mecklenburg-Western Pomerania prescribes the Dortmund Developmental Screening for Preschools (Dortmunder Entwicklungsscreening für den Kindergarten, DESK 3–6) as the instrument to monitor the development of children of the preschools involved [29]. This screening is designed to monitor the development of children in the domains of motor, linguistic and social development [34]. It helps preschool teacher to monitor the development of the children in daily situations and indicates first hints for a developmental delay. Besides the items to the four main-outcomes, the screening collects also general information about the child and the preschool. Especially important for our analysis are socio-demographic information about the child (name, age, sex, birthday, mother language, presence of disability or chronic disease and regularity of preschool-attendance). Besides that, the screening also provides information about the child’s performance at the screening and about the preschool.

In previous analyses, the test developers examine the validity of the screening instrument. These indications show that the DESK 3–6 is discriminated between children whose development was at risk according to the rating of the preschool teacher [36]. In addition, longitudinal study shows how well learning and behavior problems in second-grade students were predicted by preschool screening in the last year of kindergarten. For that, preschool teacher screened 136 six- to five-year-olds with the DESK 3–6 and the Verhaltensbeurteilungsbogen für Vorschulkinder (Behavior Assessment Test for Pre-School Children; VBV 3–6). The results show, that the DESK 3–6 allows a more reliable prediction of learning and behavioral problems in the second school year than the assessment of cognitive, language and social development of the preschool teacher [37]. According to the mentioned results, the DESK 3–6 can be considered as standardized, valid and reliable [36, 37].

The screening includes active and monitoring exercises rated on three-point scales (*yes-incomplete/partially-no* or *very often/often-sometimes-rarely/never*).

The domain social development includes tasks to assess the ability to cope with daily routines and the respecting of rules. Please see the Additional file 1 for the translated items.^2^

The amount of successfully fulfilled tasks (screening-points) in one domain is converted into age-adjusted “stanine values” (standard nine values) ranging between 1 and 9 by using the norm Tables. A stanine value of 1 (corresponding to percentile ranks 0–4) indicates reasonable findings in the dimension of social development. These children solved less tasks successfully than other children of their age. The result gives a hint for a developmental delay; further diagnosis by an external expert is suggested. A stanine value of 2 (corresponding to percentile ranks 4–11) denotes an inconclusive finding. A definite decision about a developmental delay is not possible, further observation and repetition of the DESK is recommended. Stanine values between 3 and 9 mean there is no finding (corresponding to percentile ranks 11–100) and a normal development [29].

The DESK 3–6 measurement is age-adjusted and is available in three different age versions (one for 3-year-olds, one for 4-year-olds and one for 5-to-6-year-olds). The older the child the stricter is the rating of the DESK 3–6 tasks. The norm tables are also age-specific (one norm table for children aged 3 years 0–5 months vs. 3 years 6–11 months vs. 4 years 0–5 months vs. 4 years 6–11 months vs. 5 years vs. 6 years respectively). For example: 4 years 5 months-year-old child can solve six tasks means a stanine value of 3 (no finding); one year later with the same result the child would score a stanine value of 2 (an inconclusive finding) [29, 31, 36].

### Implementation of the study

The study was approved by the Ethics Committee of the University Medicine Greifswald, Institute for pharmacology (ethic approval BB109/11). Prior to applying the DESK 3–6 staff members from every preschool were trained in how to perform the developmental screening. Subsequently, to ensure that the person conducting the screening knew the children well the screening was conducted by the familiar preschool teacher. The training was developed earlier within the framework of a pilot project [31] and was conducted by the project team. Participation in the DESK 3–6 is mandatory for every child but the parents have to provide written consent for the completed DESK-questionnaires to be shipped to the project team for evaluation.

### Sampling design and data analysis

The sample included all 3 to 6 year old children whose parents had given consent, from a total of 90 preschools receiving financial support in accordance with the federal state law for child day-care and preschools in Mecklenburg-Western Pomerania.

**Fig. 1:**
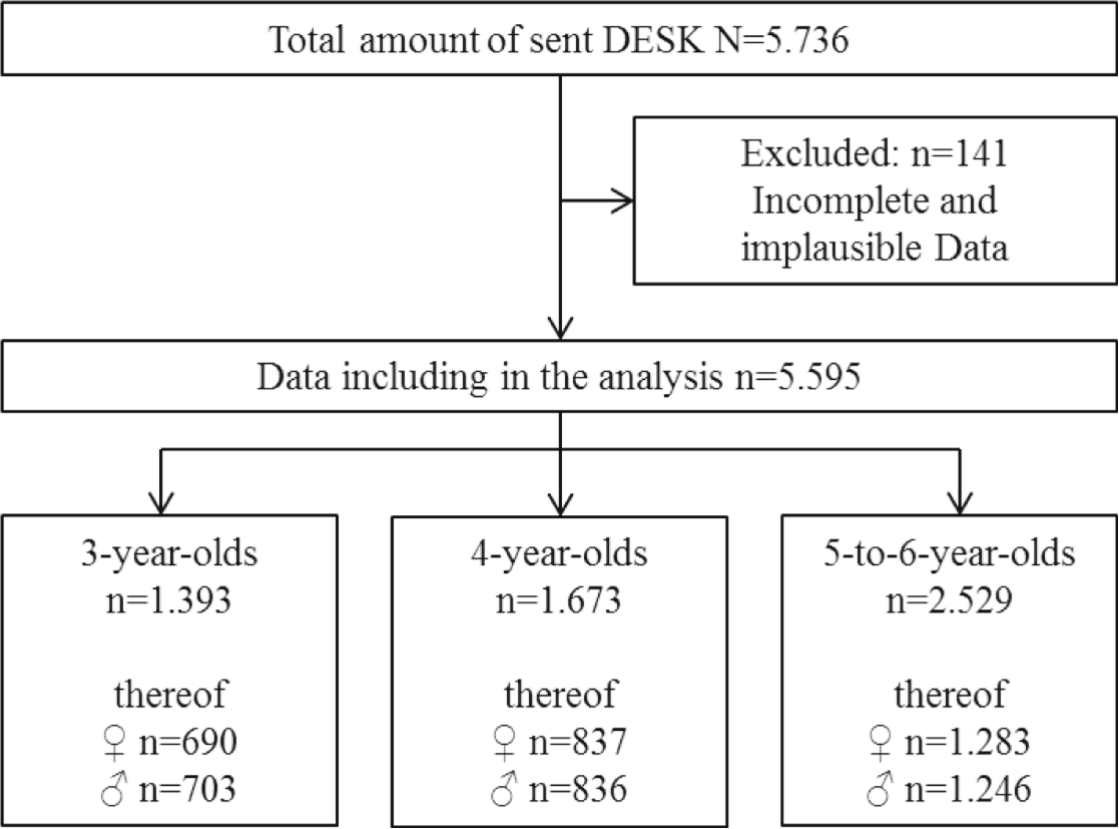
Database

After checking for completeness and plausibility, 5595 DESK screening tests were able to be included in the analysis, 141 were excluded because of missing data (see Fig. 1).

### Statistical methods

The stanine-scores were calculated using the SAS statistical software package (Version 9, SAS Institute Inc., Cary, USA). To allow comparability with the KiGGS results [6] children with a stanine of 1 and stanine of 2 in social development were combined into one category. Sex differences in stanine-values for social development were evaluated by T-tests.

We then calculated multilevel linear models (Linear Mixed Models) based on a two-level hierarchy with children’s DESK-scores nested within each individual preschool as the contextual-level predictor. Since we assumed a variation of the DESK-scores in the social development between the “DESK-day care centers”, we assumed that the intercepts vary around the overall model (random intercept model). We included the following predictor variables: (1.) sex (Coding: 1 = boys, 2 = girls), (2.) the regularity of preschool attendance (Coding: 0 = unregular attendance, 1 = regular attendance), (3.) the native language (Coding: 1 = German, 2 = Non-German), (4.) presence of a chronic disease or disability (Coding: 0 = no, 1 = yes), (5.) the dichotomized DESK stanine score for language and cognition (Coding: 0 = no finding (Stanine score between 3 and 9), 1 = reasonable findings or inconclusive finding (Stanine score of 1 or 2)), (6.) the dichotomized DESK stanine score for fine motor (Coding: 0 = no finding (Stanine score between 3 and 9), 1 = reasonable findings or inconclusive finding (Stanine score of 1 or 2)) and (7.) the dichotomized DESK stanine score for gross motor (Coding: 0 = no finding (Stanine score between 3 and 9), 1 = reasonable findings or inconclusive finding (Stanine score of 1 or 2)).

The dichotomized DESK stanine score in the domain social development served as the outcome variable (Coding: 0 = no finding (Stanine score between 3 and 9), 1 = reasonable findings or inconclusive finding (Stanine score of 1 or 2)).

Separate multilevel models were fitted for each age group, because the DESK 3–6 offers different age versions and age-adjusted norm tables. Besides that the findings separately for each age cohort are more detailed and allow individualized age-specific interventions.

All inference statistics were based on an α error probability of 0.05.

All analyses were performed with SPSS (Version 22, IBM, Armonk, USA).

## Results

### Description of the study sample

*N* = 5595 children with complete DESK-results were included in the analysis. *N* = 2785 were male (49.8%) and *N* = 2810 were female (50.2%) (see Fig. 1). Most of the included children are German native speaker (*n* = 2363). Just 83 children have a chronic disease or disability (see Table 1). One thousand one hundred forty-six children (20.83%) of our sample show delays in language and cognition, 441 children (8.04%) show delays in the domain of fine motor and 192 children (3.50%) show delays in the domain gross motor (see Table 1).

**Table 1:** Descriptive characteristics of the study sample (*N* = 5595)

	3-year-olds n (%)	4-year-olds n (%)	5/6-year-olds n (%)
**Sex**			
female	690 (49.5)	837 (50.0)	1283 (49.3)
male	703 (50.5)	836 (50.0)	1246 (50.7)
**Native Language**			
German	1313 (96.5)	1546 (95.6)	2359 (95.4)
Other	47 (3.5)	72 (4.4)	113 (4.6)
**Presence of chronic disease/ disability**			
Yes	17 (2.0)	57 (5.5)	83 (4.6)
No	838 (98.0)	987 (94.5)	1737 (95.4)
**Regularity of preschool attendance**			
Regular	1297 (96.6)	1559 (95.8)	2398 (95.9)
Irregular	45 (3.4)	69 (4.2)	102 (4.1)
**Stanine score in language/ cognition**			
Stanine score = 1	252 (18.8)	462 (28.3)	432 (17.2)
Stanine score = 2	212 (15.8)	134 (8.2)	260 (10.4)
Stanine score = 3–9	892 (65.4)	1038 (63.5)	1820 (72.5)
**Stanine score in fine motor**			
Stanine score = 1	90 (6.7)	146 (8.9)	205 (8.2)
Stanine score = 2	78 (5.8)	132 (8.1)	247 (9.8)
Stanine score = 3–9	1173 (87.5)	1356 (83)	2.060 (82)
**Stanine score in gross motor**			
Stanine score = 1	49 (3.7)	39 (2.4)	104 (4.1)
Stanine score = 2	84 (6.3)	86 (5.3)	209 (8.3)
Stanine score = 3–9	1208 (90)	1509 (92.3)	2199 (87.6)

*Note.* missing data are not included

### Prevalence of social developmental delays

Table 2 shows that for 539 (9.6%) children there are reasonable findings with regard to social skills (stanine value = 1). The results for a further 348 children (6.2%) are inconclusive (stanine value = 2). The prevalence varies with age and sex. Delays with regard to social development vary between 8.5% (3-year-olds) and 10% (5-to-6-year-olds).

The statistical prevalence of developmental delays in terms of social skills differs significantly by sex. 13.8% of the boys, but only 5.5% of the girls are affected by such developmental delays (T (df = 5548.422) = − 16.652; *p* < .001, see Table 2). This result shows a medium effect size (Cohen’s d = 0.44).

**Table 2:** Prevalence of developmental delays in the social development (N = 5595)

	DESK-results						
	Reasonable finding (stanine value = 1)		Inconclusive finding (stanine value = 2)		No finding (stanine value = 3–9)		M (SD)
	***n***	***%***	***n***	***%***	***n***	***%***	
**Total**	539	9.6	348	6.2	4.708	84.2	4.86 (2.10)
**Sex**							
Female	154	5.5	135	4.8	2.521	89.7	5.31 (1.97)
Male	385	13.8	213	7.6	2.187	78.6	4.40 (2.13)
**Age**							
3 years	119	8.5	89	6.4	1.185	85.1	5.14 (2.23)
4 years	166	9.9	140	8.4	1.367	81.7	4.88 (2.15)
5/6 years	254	10.0	119	4.7	2.156	85.3	4.68 (1.98)

*Note.* DESK - Dortmund Preschool Developmental Screening DESK 3–6, M = mean, *SD* Standard deviation

### Risk factors for the social development

For 3-year-olds, the variables sex, native language, presence of a chronic disease or disability, the DESK-score in the domain language and cognition as well as the DESK-score in the domain gross motor are statistically significant predictors for the social development (see Table 3).

**Table 3:** Multilevel regression coefficients for the prediction of children’s social developmental (dichotomized DESK stanine scores)

3-year-olds (***n*** = 816)				
predictors	b	***SE*** b	***95% CI***	***p***
sex	−0.053944	0.022676	−0.098460, −0.009427	0.018
Native language	0.172885	0.067545	0.040289, 0.305481	0.011
Regularity of preschool attendance	0.002057	0.061894	−0.119439, 0.123552	0.973
Presence of a disability or chronic disease	0.206242	0.084848	0.039674, 0.372810	0.015
DESK-score in language and cognition	0.093511	0.025405	0.059371, 0.165191	< 0.0001
DESK-score in fine motor	0.031455	0.022107	0.007933, 0.124715	0.155
DESK-score in gross motor	0.111811	0.042800	0.052802, 0.236766	0.009
**4-year-olds (*n*** **= 999)**				
**predictors**	**b**	***SE*b**	***95% CI***	***p***
sex	−0.078674	0.021819	−0.121493, −0.035855	< 0.0001
Native language	0.073159	0.056233	−0.037198, 0.183515	0.194
Regularity of preschool attendance	−0.115827	0.052727	−0.219300, − 0.012355	0.028
Presence of a disability or chronic disease	0.107762	0.052266	0.005192, 0.210332	0.039
DESK-score in language and cognition	0.093928	0.022850	0.058307, 0.151311	< 0.0001
DESK-score in fine motor	0.059525	0.022037	0.028812, 0.122978	0.007
DESK-score in gross motor	0.071763	0.030772	0.030968, 0.166301	0.020
^1^**5/6-year-olds (*n*** **= 1761)**				
**predictors**	**b**	***SE*b**	***95% CI***	***p***
sex	−0.067084	0.015158	−0.096815, −0.037353	< 0.0001
Native language	0.043453	0.036386	−0.027914, 0.114820	0.233
Regularity of preschool attendance	−0.047334	0.036277	−0.118487, 0.023818	0.192
Presence of a disability or chronic disease	0.183973	0.038899	0.107679, 0.260266	< 0.0001
DESK-score in language and cognition	0.037937	0.010529	0.022020, 0.065357	< 0.0001
DESK-score in fine motor	0.073800	0.020807	0.042469, 0.128245	< 0.0001
DESK-score in gross motor	0.067903	0.022587	0.035379, 0.130326	0.003

Notes

Outcome variable: dichotomized DESK-Score in the social development (Coding: 0 = no finding (Stanine score between 3 and 9), 1 = reasonable findings or inconclusive finding (Stanine score of 1 or 2))

Predictor variables: (1.) sex (Coding: 1 = boys, 2 = girls), (2.) the regularity of preschool attendance (Coding: 0 = unregular attendance, 1 = regular attendance), (3.) the native language (Coding: 1 = German, 2 = Non-German), (4.) presence of a chronic disease or disability (Coding: 0 = no, 1 = yes) and (5.) the dichotomized DESK stanine score for language and cognition (Coding: 0 = no finding (Stanine score between 3 and 9), 1 = reasonable findings or inconclusive finding (Stanine score of 1 or 2)) (6.) the dichotomized DESK stanine score for fine motor (Coding: 0 = no finding (Stanine score between 3 and 9), 1 = reasonable findings or inconclusive finding (Stanine score of 1 or 2)) (7.) the dichotomized DESK stanine score for gross motor (Coding: 0 = no finding (Stanine score between 3 and 9), 1 = reasonable findings or inconclusive finding (Stanine score of 1 or 2))

The predictor variables sex, regularity of preschool attendance, presence of a chronic disease or disability, the DESK-score for language and cognition, the DESK-score for fine motor and the DESK-score for gross motor are statistically significant for the social development of 4-year-olds (see Table 3).

Referring to 5/6-year-olds, the variables sex, presence of a chronic disease or disability, the DESK-score for language and cognition, the DESK-score for fine motor and the DESK-score for gross motor are statistically significant predictors for the social development (see Table 3).

## Discussion

Possible risk factors in the social development of children and their prevalence rates are relevant for planning comprehensive activities to promote children’s development in preschool, especially those in social hotspots.

The results on developmental delays and their prevalence presented here can be considered to be representative for preschool population in the social hotspots of one German federal state. These comprehensively assessed results can be considered a reliable basis for the development of evidence-based measures. In summary, lower social skills are a frequent problem in the development of children aged 3 to 6 years in preschools in the study region: in the case of 8.5% of the 3-year-olds, 9.9% of the 4-year-olds and 10.0% of the 5-to-6-year-olds there are reasonable findings with regard to their social development.

Our previous analysis from our pilot-study revealed higher prevalence (15.4% of the children had reasonable findings with regard to their social development, 7.7% were inconclusive [3]).

Overall, results from the multilevel models clearly indicate the following target groups for comprehensive measures to promote social skills: boys, children affected by chronic diseases or disabilities, children with low DESK-scores in the area of language and cognition and children with low DESK-scores in the area of motor skills.

Similar to other research, our results also show that gender is a significant risk factor over all age cohort groups in terms of the social development of children [3, 6]. Boys have a higher risk in terms of social development than girls. Our results show that gender differences can be found from age 3 onwards and they become more frequent approaching school age [38]. The KIGGS-study confirmed that the prevalence of different negative aspects in terms of a child’s social development (for example behavioral problems, emotional problems) vary with sex [6]. Our data show the necessity of taking into account sex differences when considering preventive activities. Nevertheless, a comparison of the regression coefficients of sex differences with those for older age (3-year olds: b = − 0.0539, 4-year olds: b = − 0.0787, 5-to-6-year-olds: b = − 0.0671) indicates that such activities should be for 3-year olds in particular (or even younger children) in order to prevent the stronger sex differences currently present among the older children.

The two most obvious factors affecting a delay in the social development are reasonable findings 1) in the domain language and cognition and 2) in the domain of motor skills.

The results of this study are in line with recent literature [26]. For example, language and cognition skills are important requirements for every social interaction. In addition, interactions and relationships with others are also dependent on motor skills. If a child cannot keep up with its peers in sport activities, peers often tease them and they avoid situations in which they have to do sport [11] . Furthermore, linguistically competent children are more able to control their emotions and promote more effective social interactions and friendships [2]. Against this background, one would expect that having a native language other than German is also a significant risk factor for the social development of children in preschools. In this study, however, native language is not a major determinant. One reason may be the low percentage of non-German native speakers in Mecklenburg-Western Pomerania.

Presence of a chronic disease or disability is a risk factor for the social development over all age cohort groups. This is in line with previous results [39–41]. Children with a chronic disease or disability must be a focus for preventive activities because they are likely to be vulnerable for delays in terms of their social development. Preventive interventions in preschools make it possible to reach those children without stigmatization. Again, a comparison of the regression coefficients stratified by age (3-year olds: b = 0.2062, 4-year-olds: b = 0.1077, 5-to-6-year olds: b = 0.1839) indicates that such activities should be especially targeted as early as possible to mitigate the widening differences in this group of preschoolers.

Irregular preschool attendance is potential risk factor for 4-year-old children. A possible reason for this not being statistically significant is that children attending a preschool irregularly might compensate the lack of experiences within the preschool by being involved in alternative activities that also promote their social skills (e.g. leisure activities or activities within the family). Positive effects of preschool attendance on children’s state of health have been reported [24, 42, 43]. This may indicate that regular preschool attendance can reduce social inequalities and contribute to more equal chances for social development in the pivotal period prior to school enrolment. For this reason, greater utilization of preschools in the population and increased preschool attendance should be a focus of preventive strategies.

The presented prevalence of developmental delays in preschoolers of our sample demonstrate that early preventive activities are relevant. The transitions from family life to a preschool and from a preschool to school are periods associated with significant changes and challenges that the children have to cope with. Children should be well prepared for these developmental tasks and transitions. That is why application of screening instruments and preventive interventions should start in the early preschool years. Because of the high utilization rate of preschools in Mecklenburg-Western Pomerania these institutions provide a unique setting to promote social skills. The institution preschool reaches a high proportion of children, largely independent of their social background. Our data show that early activities are necessary to achieve equal opportunities in social development with a focus on the time before children start school. Moreover, the DESK 3–6 is an adequate way to obtain a first impression of a child’s development.

### Limitations and strengths of the present study

The DESK 3–6 is designed as a screening tool with a three-point scale. Children who carry out the tasks in an incomplete way get no points for the assessment and thus the parts of a task are not converted into stanine values. If these incomplete tasks were to be considered in the results, the prevalence might be different. Further, one needs to take into account that the parents have to give written consent for the screening results to be made available for the evaluations. Therefore, selection bias cannot be completely ruled out. Another limiting factor is the definition of social skills, which covers a variety of different aspects including peer problems and behavioral problems. This lack of a consistent definition as well as the use of different screening instruments and questionnaires reduces the comparability with other population-based studies. For further research, social and emotional skills have to be considered together because these factors affect each other intrinsically and cannot clearly be delimited. Due to different methods, the comparison of our results with other mentioned studies is generally restricted – except the results of our pilot-study [3].

Our logistic model using the three other DESK-domains as single predictors is a likely simplification of a more complex interplay between the different domains of childhood development according to the DESK-screening. In a sensitive analysis, we have re-run our model including the two-way interactions between the domains of language and cognition, fine motor and gross motor development (results not shown). These had no effect on the risk factors sex, native language, regularity of preschool-attendance and presence of a chronic disease/ disability. The parameter estimates was small and the impact on the primary DESK-domain parameters (fine motor and gross motor development, language and cognition) was overall limited and inconsistent over the age groups. In our analysis, the complex interactions between the developmental domains cannot be comprehensively determined. However, the practical consequences of this limitation are probably limited. The preschool-teachers address all domains of childhood development in their activities to promote children’s skills on both the individual and the group-level.

A strength of the study is the large sample of children aged 3–6 years in preschools (*N* = 5595). The high utilization rate of preschools allows the evaluation to include nearly every child in the study region, thus rendering the preschool population almost population-based and limiting a potential selection bias. In all preschools involved in the evaluation, the proportion of parents receiving state welfare benefits to subsidize their contribution payments for the preschools was above average. This was a precondition for the preschools to qualify for financial support according to the federal state law for child day-care and preschools in Mecklenburg-Western Pomerania. Thus, the data on the prevalence of social developmental delays and their determinants can be considered to be representative for preschools in social hotspots in Mecklenburg-Western Pomerania.

### Future prospects

The law’s goal is to provide equal opportunities for all children prior to them starting school. To evaluate this aim it is ultimately necessary to analyze children’s development with a longitudinal perspective. Moreover, it is important to obtain personal longitudinal data from children during the period in which they attend a preschool until they start school. Therefore, it is necessary to link individual data collected from the period in the preschool-time with data from the school entry examination.

## Conclusions

Our results have direct relevance for ongoing debates regarding the monitoring of developmental delays and promoting of children in preschools. Our findings provide a valid basis for a health policy decision about interventions to promote social skills taking into consideration risk factors in early childhood. All in all the federal state law for child day-care and preschools is an important legal framework to achieve equal opportunities before children start school. The state government of Mecklenburg-Western Pomerania motivation is to reduce the strong impact of social inequalities on developmental health of children. This federal state law is a good example for the monitoring of preschoolers development. It allows the implementation of early prevention to reduce the prevalence of developmental delays in early childhood.

## Supplementary information

**Additional file 1.** Translated items of the domain social development of the DESK 3-6.

## Data Availability

The datasets generated and/or analyzed during the current study are not publicly available.

## Abbreviations

DESK 3–6: Dortmund developmental screening for preschools
SGB: Sozialgesetzbuch (social code of Germany)

## Footnotes

1 Due to the voluntary character, preschools can cancel the participation in the project any time. Additionally, each year new preschools can be included in the study. Another dynamic aspect of our study is that children can be in- or excluded in the preschool as well as in our study.

2 The original screening is in German language. Please note that we translated the items in English for this publication. The English items are not validated.
