# Supplementary material for: Social developmental delays among 3 to 6 year old children in preschools in German social hotspots: results of a dynamic prospective cohort study
Source: BMC Pediatr. 2020 May 13;20:216. doi: 10.1186/s12887-020-02128-3 (PMC7222296; doi:10.1186/s12887-020-02128-3)
Supplement: Supplementary file 1 — Additional file 1. Translated items of the domain social development of the DESK 3-6. [file 12887_2020_2128_MOESM1_ESM.docx]

3-year-olds

| Spielt mit der Puppe oder dem Teddybären fürsorglich. | Plays caringly with a doll or a teddy bear. |
| --- | --- |
| Spricht von sich in der Ich-Form. | Speaks of himself in the first-person. |
| Erkennt Junge und Mädchen. | Can differ between boys and girls. |
| Wäscht sich die Hände selbstständig. | Washes his hands on his own. |
| Beurteilt das Verhalten anderer Kinder. | Judges the behavior of other children. |
| Zieht Mantel oder Jacke selbstständig aus, wenn alle Verschlüsse geöffnet sind. | Undresses coat or jacket on its own if all locks are opened. |
| Verlangt in der Regel tagsüber rechtzeitig nach der Toilette, wenn es muss. | Asks for a toilet in time, if necessary. |
| Trennt sich leicht von der Mutter/dem Vater. | Separates easily from the mother / father. |
| Spielt konstruktiv und baut, ohne gleich wieder zu zerstören. | Plays constructively and builds up something without immediately destroying it. |
| Räumt nach dem Spiel einiges weg, gegebenenfalls nach Aufforderung. | Puts away the things after playing with it, if needed after request. |

4-year-olds

| Trennt sich leicht von der Mutter/dem Vater. | Separates easily from the mother / father. |
| --- | --- |
| Spielt konstruktiv und baut, ohne gleich wieder zu zerstören. | Plays constructively and builds up something without immediately destroying it. |
| Räumt nach dem Spiel einiges weg, gegebenenfalls nach Aufforderung. | Puts away the things after playing with it, if needed after request. |
| Zieht die Schuhe richtig an, d. h. linker Schuh - linker Fuß und rechter Schuh - rechter Fuß. | Put the shoes on properly, that means left shoe - left foot and right shoe - right foot. |
| Hält sich an die Regeln altersgemäßer Spiele (Brettspiele, Kartenspiele). | Follows the rules of age-appropriate games (board games, card games). |
| Reagiert auf emotionale Äußerungen anderer Kinder angemessen. | Reacts adequately to emotional expressions of other children. |
| Übernimmt in Rollenspielen eine Rolle und behält diese bei. | Takes a role in role games and keeps them. |
| Hat zeitweilig (über einige Wochen) eine Freundschaft zu einem anderen Kind. | Builds a friendship to another child temporarily (over several weeks. |
| Versorgt sich am Tisch selbst. | Is self-catering while sitting at the table. |
| Kann Streit und Konflikte friedlich lösen. | Solves disputes or conflicts peacefully. |
| Erkennt gefährliche Situationen und verhält sich angemessen. | Detects dangerous situations and behaves appropriately. |

5/6-year-olds

| Hält sich an die Regeln altersgemäßer Spiele (Brettspiele, Kartenspiele). | Follows the rules of age-appropriate games (board games, card games). |
| --- | --- |
| Reagiert auf emotionale Äußerungen anderer Kinder angemessen. | Reacts adequately to emotional expressions of other children. |
| Übernimmt in Rollenspielen eine Rolle und behält diese bei. | Takes a role in role games and keeps them. |
| Hat zeitweilig (über einige Wochen) eine Freundschaft zu einem anderen Kind. | Builds a friendship to another child temporarily (over several weeks. |
| Kann Streit und Konflikte friedlich lösen. | Solves disputes or conflicts peacefully. |
| Erkennt gefährliche Situationen und verhält sich angemessen. | Detects dangerous situations and behaves appropriately. |
| Stellt in der Gruppe eigene Bedürfnisse vorläufig zurück. | Restores own needs temporarily in the group. |
| Wartet, bis es an der Reihe ist. | Waits until being the next in line. |
| Hört Anleitungen und Erzählungen der Erzieherin aufmerksam zu. | Listens to instructions and narratives carefully. |
| Kann mit Misserfolgen angemessen umgehen. | Is able to cope with own failures. |
| Nimmt Anweisungen der Erzieherinnen auf und führt diese aus. | Takes instructions from the educators and executes them. |
| Ist in seinem Verhalten anderen Kindern gegenüber kooperativ. | Is cooperative in his behavior towards other children. |
| Geht auf Spiel- und Kontaktangebote anderer Kinder ein. | Responds to game and contact offerings from other children. |
| Kann sich ausdauernd und konzentriert über eine längere Zeit mit einer Aufgabe beschäftigen. | Can be persistent and focused on a task for a longer time. |
